# pHLuc, a Ratiometric Luminescent Reporter for *in vivo* Monitoring of Tumor Acidosis

**DOI:** 10.3389/fbioe.2020.00412

**Published:** 2020-05-08

**Authors:** Tiffany T. Ong, Zhiwei Ang, Riva Verma, Ricky Koean, John Kit Chung Tam, Jeak Ling Ding

**Affiliations:** ^1^Department of Biological Sciences, National University of Singapore, Singapore, Singapore; ^2^Division of Surgery, Yong Loo Lin School of Medicine, National University of Singapore, Singapore, Singapore

**Keywords:** pH-sensitive ratiometric luminescent reporter, S*uperecliptic* pHluorin (SEP), Nanoluc, tumor microenvironment, acidosis, bioluminescence resonance energy transfer

## Abstract

Even under normoxia, cancer cells exhibit increased glucose uptake and glycolysis, an occurrence known as the Warburg effect. This altered metabolism results in increased lactic acid production, leading to extracellular acidosis and contributing to metastasis and chemoresistance. Current pH imaging methods are invasive, costly, or require long acquisition times, and may not be suitable for high-throughput pre-clinical small animal studies. Here, we present a ratiometric pH-sensitive bioluminescence reporter called pHLuc for *in vivo* monitoring of tumor acidosis. pHLuc consists of a pH-sensitive GFP (*superecliptic* pHluorin or SEP), a pH-stable OFP (Antares), and Nanoluc luciferase. The resulting reporter produces a pH-responsive green 510nm emission (from SEP) and a pH-insensitive red-orange 580nm emission (from Antares). The ratiometric readout (R_580__/__510_) is indicative of changes in extracellular pH (pH_e_). *In vivo* proof-of-concept experiments with NSG mice model bearing human synovial sarcoma SW982 xenografts that stably express the pHLuc reporter suggest that the level of acidosis varies across the tumor. Altogether, we demonstrate the diagnostic value of pHLuc as a bioluminescent reporter for pH variations across the tumor microenvironment. The pHLuc reporter plasmids constructed in this work are available from Addgene.

## Introduction

A hallmark of neoplastic diseases is the reprogramming of cellular energy metabolism to actively support cell proliferation (Hanahan and Weinberg, 2011). Unlike normal cells, cancer cells display an increased rate of glycolysis even under normal oxygen conditions. This “Warburg effect” leads to excessive production of lactic acid, and acidification of the tumor microenvironment, with the extracellular pH (pH_e_) dropping to as low as 6.4 (Chen et al., 2015). Tumor acidosis has been shown to promote invasion, metastasis, and drug resistance due to neutralization of weak base chemotherapeutic drugs, resulting in aggressive cancer phenotypes and ultimately, reduced patient survival (Chen et al., 2015; Corbet and Feron, 2017; Pillai et al., 2019).

Despite the significance of studying the role of pH_e_ in tumor progression, limited methods exist to monitor the pH_e_ of tumors *in vivo*. Measuring the pH of local tissues via invasive methods such as the surgical insertion of pH microelectrodes, may trigger inflammatory processes that can, by itself, alter the pH (Menkin, 1933; Chen et al., 2015). *In vivo* imaging approaches utilizing pH sensitive magnetic resonance imaging (MRI) dyes (Sun and Gregory Sorensen, 2008; Hashim et al., 2011; Chen and Pagel, 2015; Longo et al., 2016) or positron emission tomography (PET) dyes tagged to the pH-sensitive pHLIP peptide (Reshetnyak et al., 2007; Chen and Pagel, 2015) require costly equipment and lengthy image acquisition times. On the other hand, a genetically encoded pH-sensitive luminescence reporter would provide a simple and inexpensive means to study the pH_e_ of tumors *in vivo*. However, to the best of our knowledge, no such bioluminescence pH reporter exists hitherto.

S*uperecliptic* pHluorin (SEP) is a mutant of GFP that is widely used *in vitro* as a fluorescence reporter of pH, and is nearly non-fluorescent at pH 6 but brightly green fluorescent at pH 7.4 (Miesenböck et al., 1998). However, SEP is ill-suited for *in vivo* imaging due the high background autofluorescence, typically encountered during *in vivo* fluorescence imaging (Puaux et al., 2011). Due to the high background autofluorescence brought about by fluorescent probes, *in vivo* imaging is most commonly performed with luminescent reporters such as Firefly or *Renilla* luciferase reporters, and the more recent Nanoluc luciferase reporters (Schaub et al., 2015). Nanoluc reporters hold many advantages over Firefly or Renilla luciferase, being 100-fold brighter and not requiring ATP as a substrate. The ATP-free reaction allows Nanoluc to be used in the ATP-deficient extracellular space (Pfleger and Eidne, 2006; Hall et al., 2012). Thus, an ideal reporter to study the pH_e_ of tumors *in vivo* would possess the excellent pH-sensitivity of SEP and the bright extracellular luminescent signal potential of Nanoluc.

**FIGURE 1:**
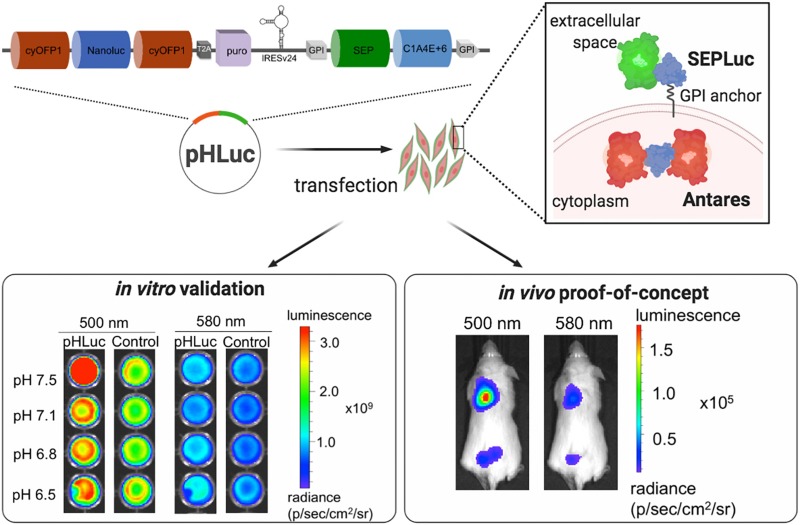
Overview of the pHLuc system and experimental workflow. The pHLuc reporter is a multicistronic cassette that comprises of cytoplasmic Antares, T2A-puromycin, a mutated internal ribosome entry site (IRES) v24, and a membrane-bound SEPLuc that utilizes a weaker variant of Nanoluc. Cells transfected with pHLuc have shown that the reporter is pH-responsive compared to a pH-stable control. Since the SEP signal is pH-dependent, the ratio of SEP to Antares (R_580__/__510_) is indicative of changes in extracellular pH. When cells stably expressing pHLuc were injected into mice, bioluminescence imaging also showed differences in the ratiometric readout. *In vivo* ratiometric images showed the capacity of pHLuc to detect extracellular pH changes. Image generated via BioRender.

Here, we describe a genetically encoded luminescence reporter, pHLuc, which combines the pH-sensitivity of SEP with the bright extracellular luminescent signal of Nanoluc to allow for the *in vivo* whole animal imaging of tumor pH_e_ (Figure 1). The pHluc system consists of two bioluminescent reporters, SEPLuc and Antares. SEPLuc is an optimized fusion of SEP and Nanoluc that is anchored to the cell surface via glycosylphosphatidylinositol (GPI). Through efficient bioluminescence resonance energy transfer (BRET) of the donor Nanoluc signal to pH-sensitive SEP, SEPLuc has a pH-sensitive green emission that peaks at 510 nm and is progressively reduced from pH_e_ 7.4 to 6. SEPLuc is bicistronically co-expressed with Antares, a cytoplasmic Nanoluc fusion that utilizes the same furimazine substrate but has pH-insensitive red-orange emission that peaks at 580 nm (Chu et al., 2016). By obtaining the bioluminescence emission ratio of SEPLuc over Antares (R_580__/__510_), the pHLuc reporter controls for pH-independent confounding variables such as changes in reporter expression, substrate availability, optical path length, movement, and growth artifacts (Thestrup et al., 2014). Through *in vivo* imaging, the R_580__/__510_ bioluminescence emission ratio of the resulting pHLuc reporter showed that human synovial sarcoma SW982 xenografts in NSG mice displayed acidosis that varied across the tumor with lower pH values observed at the center of the tumors. On the whole, pHLuc is a ratiometric bioluminescent reporter of tumor pH that allows for the non-invasive monitoring of tumor acidosis in mice via IVIS Spectrum whole animal imaging.

## Results

### Engineering a SEP-Nanoluc Fusion for Bioluminescence pH Sensing

**FIGURE 2:**
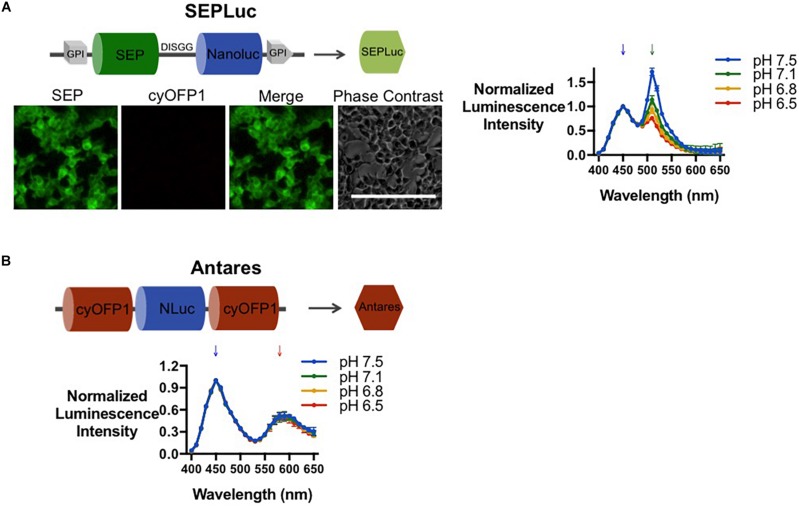
Characterization of the Nanoluc-fluorescent protein fusions expressed in HEK293T cells. **(A)** Microscopy and luciferase assay of SEPLuc (*n* = 2). **(B)** Luciferase assay of Antares (*n* = 2). Hereon, SEPLuc and Antares will be depicted as green and red shapes as indicated above (diagrams not drawn to scale). Downward arrows on spectral scan values indicate the wavelengths at which peaks are expected (450 nm for Nanoluc, 510 nm for SEP, and 580 nm for R/OFPs). All luminescence values were normalized to the Nanoluc emission (450 nm). Error bars are SD. Scale bars = 200 μm.

To develop a ratiometric pH-sensitive luminescent reporter, we first fused Nanoluc downstream of SEP through a flexible, five-residue linker sequence (Asp-Ile-Ser-Gly-Gly). This linker was recently described to optimize the folding of the two proteins (Nanoluc and eGFP) while maintaining close proximity for efficient energy transfer via BRET (Schaub et al., 2015). To constitutively express this fusion construct as a membrane protein, the N- and C-termini signals of the GPI anchor sequence were fused to the 5′ and 3′ ends of the fusion construct, respectively; henceforth, this membrane-bound SEP-Nanoluc fusion is termed SEPLuc. This initial construct was transiently transfected into HEK293T cells. Fluorescence microscopy confirmed the localization of SEP to the cell membrane (Figure 2A, left). Bioluminescence assays confirmed the capacity of SEPLuc to detect pH_e_ change *in vitro*. As the pH of the MOPS buffer was gradually decreased from 7.5 to 6.5, we observed a progressive reduction in the 510 nm emission (Figure 2A, right).

### Co-expression of SEPLuc With Antares Generates a Ratiometric Signal

Next, we attempted to identify a suitable pH-insensitive red-orange bioluminescent Nanoluc reporter that can be co-expressed with SEPLuc for *in vivo* ratiometric sensing. A red-orange bioluminescent partner is necessary because the 460 nm emission of Nanoluc is too blue-shifted for detection by *in vivo* imaging systems such as the IVIS Spectrum, which can only resolve luminescent emissions with wavelengths between 490 to 850 nm. Given that the signal of SEPLuc started to diminish at around 550 nm onward (Figure 2A), candidate ratiometric partners including the previously described OgNluc, ReNL, and Antares Nanoluc fusion reporters, which produce bioluminescent emission peaks at 572 nm (Schaub et al., 2015), 585 nm (Suzuki et al., 2016), and 584 nm (Chu et al., 2016), respectively, were tested. Among these red-orange reporters, Antares, which consists of cyOFP1s flanking the Nanoluc, displayed the highest BRET-efficiency and the most red-shifted emission (Supplementary Figure 1A). Antares also retained the most prominent 510 and 580 nm emission peaks when co-transfected with the SEPLuc plasmid (Supplementary Figure 1B) and had a 580 mm emission that was insensitive to changes in pH_e_ (Figure 2B). Thus, Antares was chosen as the pH-independent ratiometric partner for SEP-Nanoluc, for further optimization.

### Iterative Optimization of the Signal Ratios of SEPLuc and Antares During Bicistronic Co-expression

**FIGURE 3:**
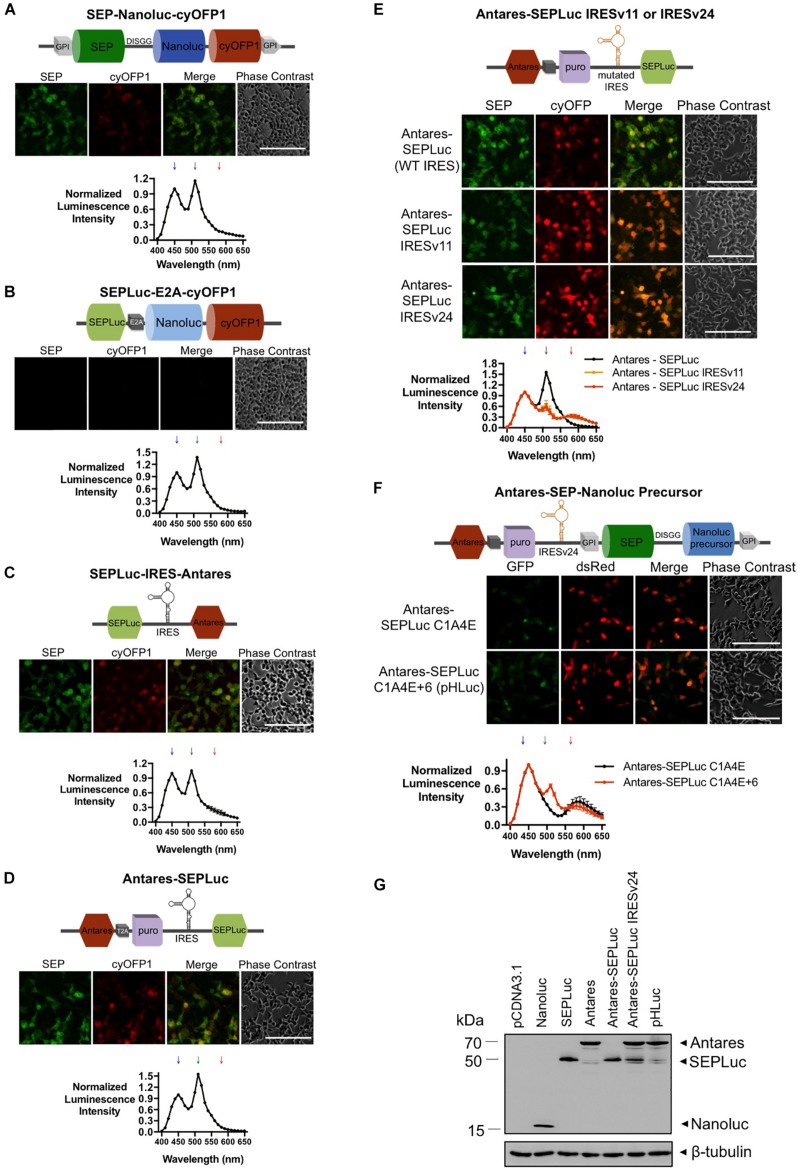
Iterative optimization of the SEPLuc and Antares bioluminescence signal ratios. **(A)** cyOFP1 was inserted into the existing membrane-anchored SEPLuc fusion via the 5-amino acid linker (*n* = 2). **(B)** cyOFP1 was fused to another Nanoluc sequence, then linked downstream of SEPLuc via E2A (*n* = 2). **(C)** Antares was cloned downstream of SEPLuc and IRES (*n* = 2). **(D)** The Antares-SEPLuc construct. Antares is followed by T2A and puromycin (colored purple), then by IRES and SEPLuc. (*n* = 3). **(E)** Comparison of the Antares-SEPLuc constructs utilizing wildtype IRES and the mutant variants IRESv11 and IRESv24 (*n* = 3). **(F)** Comparison of the Antares-SEPLuc IRESv24 constructs utilizing two weaker Nanoluc precursors, C1A4E and C1A4E+6, transiently expressed in SW982 cells (*n* = 2). **(G)** Western blot of whole cell lysates from SW982 transfected with vector control, Nanoluc, SEPLuc, Antares, Antares-SEPLuc, Antares-SEPLuc IRESv24, and pHLuc. All constructs were transfected into a model cell line, HEK293T for initial assessment of their expression, unless stated otherwise. Downward arrows on spectral scan values indicate the wavelengths at which peaks are expected (450 nm for Nanoluc, 510 nm for SEP, and 580 nm for Antares). Luciferase assay values were normalized to the Nanoluc emission at 450 nm. Error bars are SD.

Our initial reporter prototype was a simple C-terminal fusion of cyOFP1 to SEPLuc (termed NC3) via the same Asp-Ile-Ser-Gly-Gly linker used in Antares (Chu et al., 2016). While cyOFP1 was faintly visible under the microscope (Figure 3A, top), we were unable to detect the 580 nm emission peak corresponding to cyOFP1 in the luciferase assay (Figure 3A, bottom). We surmised that this may be due to the fusion of SEPLuc to cyOFP leading to protein misfolding. To overcome this, the Nanoluc-cyOFP1 fusion was separated from SEPLuc via an E2A cleavage peptide (Liu et al., 2017). However, neither of the fluorescent proteins was detected via microscopy (Figure 3B, top), although a peak corresponding to SEPLuc was detected in luciferase assay (Figure 3B, bottom). Since the E2A cleavage peptide failed, we co-expressed Antares downstream of SEPLuc via an encephalomyocarditis (EMCV) internal ribosome entry site (IRES) sequences. Microscopy showed the presence of both SEP and Antares (Figure 3C, top), but Antares was not detectable in luciferase assay (Figure 3C, bottom). Since IRES-dependent expression of the second gene may be significantly lower (Mizuguchi et al., 2002), we cloned SEPLuc downstream of the IRES to maximize the signal output at 580 nm. The Antares-SEPLuc construct also contains a T2A-puromycin fusion incorporated between Antares and SEPLuc to improve antibiotic selection of stably transfected cells. Microscopy detected both SEP and Antares (Figure 3D, top), but the Antares signal was again absent in the luciferase assay (Figure 3D, bottom).

To further improve the Antares signal relative to that of SEPLuc, we first reduced the efficiency of the IRES in the Antares-SEPLuc construct. We selected IRESv11 and IRESv24, which were previously described to reduce translation efficiency to 24 and 0.34%, respectively (Koh et al., 2013). Microscopy and luciferase assay data showed decreased expression of SEPLuc (Figure 3E). Next, we chose two weaker Nanoluc precursors that were derived from *Oplophorus gracilirostris* luciferase (Oluc-19): (1) C1A4E, which has eight mutations over Oluc-N166R (a more stable variant of Oluc-19), and (2) a mutant we dubbed C1A4E+6, which has six amino acid changes over C1A4E that contribute to enzyme stability (Hall et al., 2012). Both microscopy and luciferase assays of SW982 cells showed that C1A4E dimmed the SEPLuc signal excessively, while C1A4E+6 resulted in detectable signal at both 510 and 580 nm (Figure 3F). We therefore named this optimized final construct as pHLuc, (Antares-SEPLuc C1A4E+6). Western detection confirmed the presence and size of Antares (70 kDa) and SEPLuc (50 kDa), and showed a decreased expression of SEPLuc upon mutation of the IRES and the Nanoluc (Figure 3G).

### pHLuc Can Detect pH_e_ Changes *in vitro*

**FIGURE 4:**
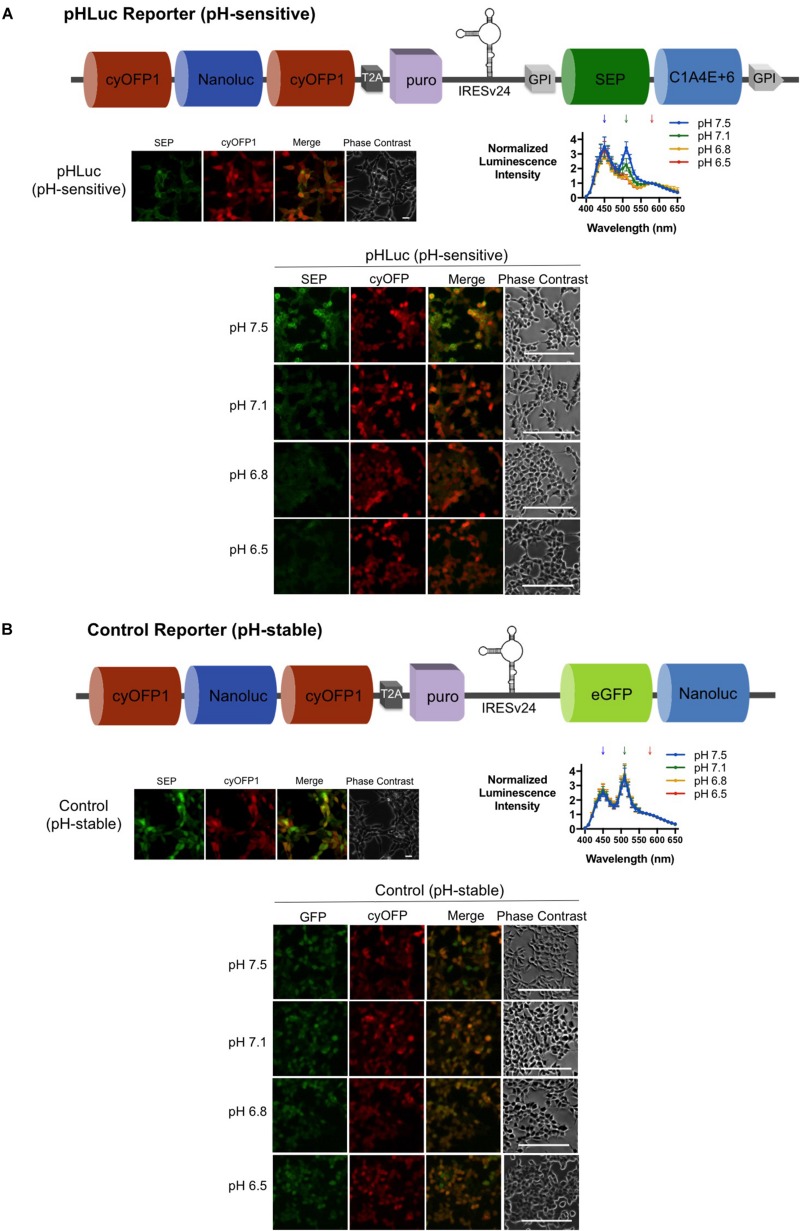
*In vitro* validation assays for SW982 cells stably expressing **(A)** pHLuc and **(B)** the pH-stable Control reporters. **(A)** Schematic diagram of pHLuc (top). Confocal microscopy of SW982 cells expressing pHLuc showing correct protein localization, scale bars = 20μm (left). Luciferase assay and microscopy of pHLuc-expressing SW982 cells subjected to different pH levels of MOPS buffers, scale bars = 200 μm (right and bottom). **(B)** Schematic diagram of the pH-stable Control (top). Confocal microscopy of SW982 cells expressing the Control reporter showing correct protein localization, scale bars = 20 μm (left). Luciferase assay and microscopy of Control reporter-expressing SW982 cells subjected to different pH levels of MOPS buffers, scale bars = 200 μm (right and bottom). Downward arrows on spectral scan values indicate the wavelengths at which peaks are expected (450 nm for Nanoluc, 510 nm for SEP, and 580 nm for Antares). Luciferase assay values were normalized to the Nanoluc emission at 450 nm. Error bars are SD.

**FIGURE 5:**
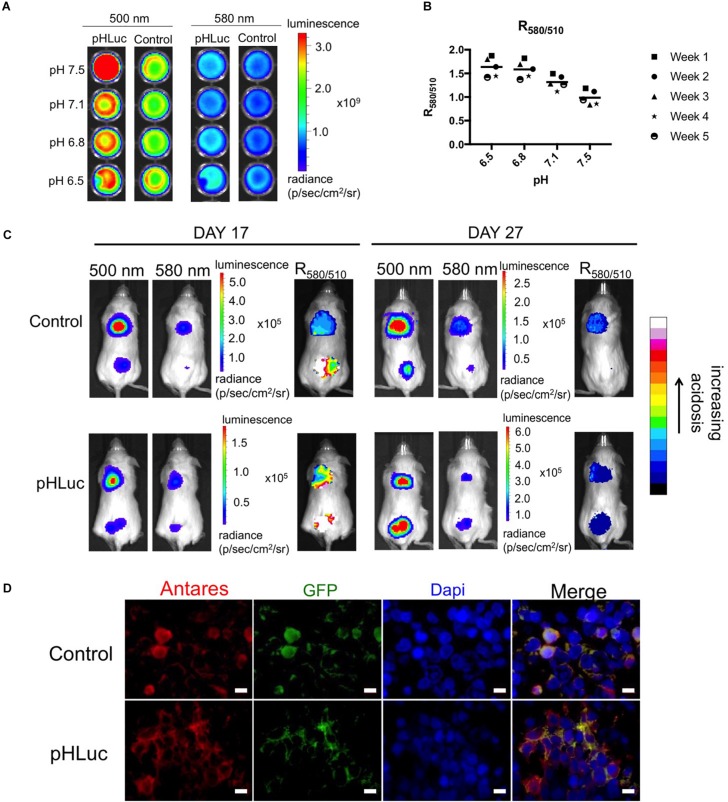
Representative bioluminescence images of pHLuc *in vitro* and *in vivo*. **(A)** Plated SW982 cells expressing the pHLuc and Control reporters, imaged under the 500 and 580 nm filters of IVIS. **(B)** R_580__/__510__*pHLuc*_ normalized with R_580__/__510__*Control*_ of stably expressing SW982 cells. The cells were imaged using the IVIS once a week. Images ratios were obtained using ImageJ. **(C)** Representative NSG mice (one for pHLuc and one for control) injected with SW982 cells expressing the respective reporters, were imaged under the 500 and 580 nm filters of IVIS on days 17 and 27. The ratiometric images (right panel of each time point) were obtained via ImageJ. **(D)** Fluorescence immunohistochemical analysis of representative tumor sections for SEP/eGFP (green) and Antares (red) expression. Nuclei (blue) were stained with Dapi. Analyses were done at the endpoint when animals were sacrificed. Scale bar = 10 μm.

To investigate whether the pHLuc reporter would produce a ratiometric signal in response to pH_e_, we generated SW982 cells that stably expressed either the pHLuc reporter or a pH-stable Control reporter that utilizes eGFP instead of SEP (full construct shown at Figures 4A,B, top). The SEPLuc and Antares components of pHLuc were expected to localize within the cell membrane and cytoplasm, respectively; while the eGFP and the Antares components of the pH-stable control were both expected to localize within the cytoplasm. This was consistent with the widefield microscopy results (Figures 4A,B, left). When subject to a decrease in pH_e_ from 7.5 to 6.5, the pHLuc-expressing cells displayed a progressive decrease in their bioluminescence and fluorescence emission intensity at 510 nm while the emission intensity at 580 nm remained constant (Figure 4A, right and bottom). On the other hand, the bioluminescence and fluorescence emission spectra of cells expressing the pH-stable control remained constant within the same pH range (Figure 4B, right and bottom). This pHe-dependent ratiometric bioluminescence signal was also detectable with the plated cells that were imaged with the IVIS spectrum (Figure 5A). Finally, the R_580__/__510_ (emission at 580 nm divided by emission at 510 nm) of the pHLuc reporter was further divided by the R_580__/__510_ of the pH-stable control to obtain the R_580__/__510__*pHLuc*_/R_580__/__510__*Control*_, which showed the expected trend of increasing values as the pH_e_ was reduced from 7.5 to 6.5 (Figure 5B).

### pHLuc Detected Variations in pH_e_ Across SW982 Tumor Xenografts in Mice

As a proof-of-concept, we tested the reporters *in vivo* in NSG mice. Figure 5C shows representative images of mice injected with the SW982 cells expressing the respective reporter. The 500 and 580 nm images show that the signal is brightest at the center of the tumor. The images showing R_580__/__510_ displayed a gradient of colors within a representative tumor, indicating a difference in pH distribution within the tumor, likely due to hypoxia and greater changes in cell metabolism in some areas. Ratiometric images of mice injected with pHLuc-expressing cells also displayed comparatively greater ratio variability, and hence pH, compared to the mice injected with control cells. Immunohistochemistry of tumor sections showed that cells that express SEP (for pHLuc tumors) or eGFP (for control tumors) also co-express Antares. The difference in localization of SEP and eGFP can also be seen (Figure 5D). Altogether, the data from the pHluc reporter suggests that the pH_e_ varies across SW982 tumors in NSG mice.

## Discussion

Most cancer cells have been reported to have pH_*i*_ of around 7.4, and pH_e_ of around 6.7–7.1 (Webb et al., 2011). This reversed pH gradient, often referred to as tumor acidosis, is now being considered a hallmark of invasive cancers (Chiche et al., 2010). Given the significant role of pH_e_ in tumor progression, we present a ratiometric luminescent reporter called pHLuc that allows for live imaging of pH change with tumor progression. The pHLuc reporter detected variations in pH_e_ across SW982 tumor xenografts in NSG mice, a finding that is consistent with other reporter systems (Anemone et al., 2019).

The initial optimizations of the pHLuc reporter utilized only cyOFP1, cloned as a fusion to SEP-Nanoluc and separately with its own Nanoluc in a bicistronic cassette. In both instances, the cyOFP1 signal was too low to be detected via microscopy and luciferase assay. In succeeding optimizations, the full Antares construct (consisting of cyOFP1s flanking the Nanoluc) was cloned upstream of SEP-Nanoluc, resulting in a detectable signal emission corresponding to Antares. However, our preliminary optimizations consistently showed that the SEP signal was higher than that of Nanoluc only, and previous studies have shown that the signal of Antares relative to Nanoluc only, is weaker (Chu et al., 2016). This may explain why the emission of Antares, although apparent, was weaker and was masked by the very strong signal of SEP. Thus, for luciferase assay data of the initial constructs (Figures 3A–E) only showed the normalized intensity because the cyOFP1 or Antares signal peak was not visible, and we were unable to obtain signal ratios. To further improve the signal of Antares in the pHLuc reporter, IRES was employed and mutated to modulate the expression of the SEP-Nanoluc, and a weaker variant of the Nanoluc was fused to SEP to reduce the green signal output. *In vitro* validation assays demonstrated the functionality of the reporter, and *in vivo* experiments showed the capacity of pHLuc to monitor pH distribution within a tumor.

To the best of our knowledge, the pHLuc reporter is the first genetically encoded pH-sensitive luminescent reporter that would provide a simple and inexpensive means to study the pH_e_ of tumors *in vivo*. An advantage of utilizing BRET for pHLuc is that the reporter ultimately combines the benefits of the bright fluorophore signal and the low background of a luciferase (Dale et al., 2019; Kobayashi et al., 2019). Moreover, this reporter provides the advantage of dual-color imaging with the use of a single furimazine substrate, and allows for the monitoring of pH changes in small rodents. Previous luminescent reporters based on firefly luciferase (Gabriel and Viviani, 2014) were only applicable for measuring intracellular pH as firefly luciferase requires ATP found in the cytoplasm as a substrate. By using Nanoluc, which does not require ATP and is active in the extracellular space, the pHLuc reporter is able to report on the pH_e_ of tumors *in vivo*.

Using a genetically encoded reporter such as pHLuc is preferable to measuring the pHe of tumors *in vivo* via the surgical insertion of pH microelectrodes, which may trigger inflammatory processes that can by itself, alter the pH (Menkin, 1933; Chen et al., 2015). Other *in vivo* imaging approaches such as pH-sensitive MRI dyes (Sun and Gregory Sorensen, 2008; Hashim et al., 2011; Chen and Pagel, 2015; Longo et al., 2016) or PET dyes tagged to the pH-sensitive pHLIP peptide (Reshetnyak et al., 2007; Chen and Pagel, 2015), all require lengthy image acquisition times and costly MRI or PET imaging systems that are not as widespread as the IVIS Spectrum *In Vivo* Imaging System (PerkinElmer). These dye based approaches also suffer from poor uptake of the dye into the tumor whereas the genetically encoded pHluc reporter is expressed throughout the tumor (Anemone et al., 2019). A current disadvantage of luminescent reporters such as the pHLuc reporter is that it can only be used to acquire 2D images while PET- or MRI-based approaches have 3D acquisition capabilities. Nevertheless, improvements in bioluminescent *in vivo* imaging technologies could potentially further enhance the sensitivity and resolution of the pHLuc reporter.

In our experiments, the pHLuc reporter did not achieve a high enough signal-to-noise ratio for us to obtain quantitative measurements via comparisons with *in vitro* pH standards. The problem of a low signal was particularly pronounced for the red emission peak of the Antares reporter (Figure 2B). Even in our final reporter design (the IRESv24 and C1A4E+6 construct or pHLuc) the red emission peak of Antares was still significantly weaker than the green emission peak of pHLuc (Figure 3F). The red emission of Antares in the dual color construct was only achieved after multiple iterative mutations to reduce the expression level and activity of SEPLuc, which resulted in a weaker ratiometric signal overall (Figure 3). The development of a luciferase with a brighter red emission relative to Antares and its integration into the pHLuc reporter system would no doubt lead to a reporter with a better signal-to-noise ratio. This reporter would also potentially allow for pH quantification by comparing the *in vivo* ratiometric signal to the ratios obtained from *in vitro* pH standards such as those described in Figures 5A,B.

Here, we describe the generation of pHLuc, a ratiometric bioluminescent reporter of tumor pH that allows for the non-invasive monitoring of tumor acidosis in mice via IVIS whole animal imaging. Alternative approaches such as pH-sensitive MRI dyes (Sun and Gregory Sorensen, 2008; Hashim et al., 2011; Chen and Pagel, 2015; Longo et al., 2016) or PET dyes tagged to the pH-sensitive pHLIP peptide (Reshetnyak et al., 2007; Chen and Pagel, 2015) do work well and have been described in the literature. However, these approaches require costly MRI and PET instrumentation. Our aim here was to develop a bioluminescent reporter that can be acquired with the more commonly available IVIS spectrum imaging system. While this study focused on bioluminescence imaging, the pHLuc reporter system could also potentially be adapted to whole body fluorescence imaging approaches depending on the instrumentation available to the researcher (Leblond et al., 2010). The pHLuc reporter could potentially have applications in determining the relationship of chemotherapy and cancer regression by monitoring tumor microenvironment changes in pH (Mordon et al., 1994; Düwel et al., 2017; Jo et al., 2017). Specifically, our pHLuc reporter could be exploited for preclinical testing of the efficacy of cancer chemotherapeutics *in vivo*, in cancer-bearing mice models. For example, drugs such as doxorubicin and mitoxantrone have reduced cytotoxicity in acidic pH, while drugs such as chlorambucil and 5-fluorouracil have higher cytotoxicity at acidic pH (Jo et al., 2017). The administration of bicarbonate bases and proton pump inhibitors have also been found to reduce tumor acidosis, growth and metastasis in various mouse models (Estrella et al., 2013; Jin et al., 2014; Xie et al., 2014). This suggests the diagnostic value of pHLuc in reporting differential pH in the tumor microenvironment, further enhancing the potential for pre-clinical drug development and treatments *in vivo*.

## Materials and Methods

### Fusion Protein Construction

The DNA sequence consisting of the GPI anchor, SEP, DISGG linker and Nanoluc was generated via DNA synthesis (Integrated DNA Technologies, Singapore). A 5′ primer encoding *Xba*I and a 3′ primer encoding *Not*I were used to generate a whole DNA fragment of the said sequence, and this was ligated into the pGEM^®^-T Easy vector (Promega). The insert was then subcloned into pCDH-EF1-MCS-T2A-Puro (CD520A-1; Systems Biosciences) via *Xba*I and *Not*I restriction enzyme digestion. pNCS-Antares (Addgene plasmid # 74279), pcDNA3.1(+)IRES GFP (Addgene plasmid # 51406), pCDNA3.1(+)ReNL (Addgene plasmid # 85203), and pRetroX-Tight-MCS_PGK-OgNLuc (Addgene plasmid # 70186) were generous gifts from Drs. Michael Lin, Kathleen L. Collins, Takeharu Nagai, and Antonio Amelio, respectively. To generate the complete pHLuc fusion protein in the pCDNA3.1(+) vector backbone (Thermo Fisher Scientific), the homologous recombination cloning protocol based on Jacobus and Gross (2015) was used. Briefly, primers containing around 20 base pairs of overlapping sequences were designed to amplify the relevant sequences from the aforementioned plasmids (using Q5 Hi-Fidelity DNA Polymerase; NEB), such that the PCR products amplified from the separate plasmids can easily recombine into a complete pCDNA3.1(+) vector backbone (with the neomycin sequence deleted) containing the full pHLuc sequence when transformed into *Escherichia coli*. PCR products were digested with 10 units of *Dpn*I (Thermo Fisher Scientific) overnight and were purified using the Wizard SV Gel and PCR Clean-Up System (Promega). At least 100 ng of each purified fragment was then transformed into 80 μL of TOP10 chemically competent *E. coli.* Screening was done via PCR using the GoTaq^®^ Green Master Mix (Promega). Positive clones were propagated in LB broth containing ampicillin (Merck) diluted to a working concentration of 100 μg/mL, and plasmids were extracted using PureYield^TM^ Plasmid Miniprep Kit (Promega). The pHluc reporter plasmids described here are deposited with Addgene and are available upon request, from Addgene (Supplementary Table 1).

### Microscopy

Human embryonic kidney 293T (HEK293T) cells were cultured in Dulbecco’s Modified Eagle Medium (DMEM; Gibco) supplemented with 10% (v/v) fetal bovine serum (FBS) (HyClone) and 1% penicillin-streptomycin (Gibco), at 37°C with 5% CO_2_. The cells were seeded in 24-well plates at least 4 h prior to transfection using TurboFect^TM^ transfection reagent (Thermo Fisher Scientific), and were assayed the following day. Briefly, cells were first observed using an Axio Observer inverted microscope (Zeiss) under phase contrast as well as eGFP and dsRed filters to verify the presence of SEP and Antares. Consistent exposure times (3–10 s) were used in taking images under both eGFP and dsRed filters.

### *In vitro* Bioluminescence Characterization Via Luciferase Assay

Transfected cells were detached with 2 mM ethylenediaminetetraacetic acid (EDTA) with 2% FBS in sterile phosphate buffer saline (PBS) (Supplementary Figure 2), and around 150,000 cells were pelleted. The pellet was resuspended in 100 μL of 25 mM 3-morpholinopropane-1-sulfonic acid (MOPS) saline buffer (adjusted to pH 7.5, 7.1, 6.8, and 6.5 using 6 M HCl) containing the Nanoluc substrate (Promega) diluted 1:1000, and dispensed into a black clear-bottom 96-well plate. Luminescence was determined using the Synergy^TM^ MX Microplate Reader (BioTek) by obtaining the spectral scan from 400–650 nm (10-nm step increments; integration time 1 s). Readings were taken one well at a time, with the substrate added to the cells just before reading, to minimize variability. Additionally, a blank reading (substrate only) was obtained using the same plate reader settings. Raw plate reader values were subtracted with the blank values, then normalized with the value corresponding to either Nanoluc (at 450 nm) or Antares (at 580 nm).

### Western Blot

SW982 human synovial sarcoma cells were cultured in Roswell Park Memorial Institute (RPMI 1640; Gibco) medium supplemented with 10% HyClone^TM^ FBS (GE Healthcare) and 1% penicillin-streptomycin (Gibco), at 37°C with 5% CO_2_. The cells were seeded in 6-well plates at least 6 h prior to transfection using the ViaFectTM transfection reagent (Promega). One day after transfection, cells were harvested and lysed. Total cell lysates were resolved on a denaturing 10% SDS-PAGE gel, and blotted onto a PVDF membrane using the Trans-Blot Turbo Transfer system (Bio-Rad). Samples were then probed with (1) Nanoluc antibody (R&D Systems; MAB100261), resuspended to 306 μg/mL and diluted to 1:200 with 5% milk in TBST, and (2) β-tubulin antibody (Cell Signaling, # 2146), which was supplied in a concentration of 100 μg/mL, and diluted to 1:1000 with the 5% bovine serum albumin (BSA) in TBST. The samples were then probed with the appropriate secondary antibody: (1) α-mouse secondary antibody diluted to 1:2500 in blocking solution for Nanoluc, and (2) α-rabbit secondary antibody diluted to 1:2500 in blocking solution for β-tubulin. The membrane was incubated in substrate ECL HRP substrate (Advansta), and chemiluminescent signals were obtained using the ImageQuant LAS 4000 mini (GE Healthcare Life Sciences).

### Generation of Stably Expressing SW982

SW982 human synovial sarcoma cells were cultured in Roswell Park Memorial Institute (RPMI 1640; Gibco) medium supplemented with 10% HyClone^TM^ FBS (GE Healthcare) and 1% penicillin-streptomycin (Gibco), at 37°C with 5% CO_2_. The cells were seeded in 6-well plates at least 6 h prior to transfection using the ViaFect^TM^ transfection reagent (Promega). One day after transfection, the media was removed and replaced with RPMI 1640 containing puromycin dihydrochloride (Thermo Fisher Scientific) diluted to a final concentration of 5 μg/mL. The medium containing the selection antibiotic was changed every 3–4 days for 2 weeks, after which the puromycin concentration was lowered to 2 μg/mL for culture maintenance.

### Subcutaneous Tumor Xenografts

All mice experiments were performed under guidelines and protocols (R16-1568) approved by the NUS Institutional Animal Care and Use Committee. Each 6-week old NOD.Cg-Prkdcscid Il2rgtm1Wjl/SzJ (NSG) mouse (InVivos, Singapore) was anaesthesized using 2% isoflurane (Attane), and injected subcutaneously at the dorsal side with 5.0 × 10^5^ and 1.0 × 10^6^ SW982 cells resuspended in 100 μL of sterile Hank’s balanced salt solution (HBSS; Gibco).

### *In vitro* pH Calibration

For pH calibration using the IVIS, cells stably expressing both pHLuc and the control were pelleted and resuspended in MOPS buffer of varying pH levels as previously described. The bioluminescence output was taken with the following settings: excitation block, emission 500 and 580 nm (20 nm bandpass filter), aperture f/2, exposure time 1 s, binning 4, and field of view 12.5 cm. As with all luciferase assays done in this study, the substrate was added to the cells only right before the spectral scan. Samples were done in triplicates, and luciferase readings were taken around 1 week apart for at least 4 weeks to ensure consistency of the ratios even as cells are cultured over time.

### *In vivo* Bioluminescence Imaging

*In vivo* imaging experiments were performed with the IVIS Spectrum (Perkin Elmer). Mice were anaesthesized using 2% isoflurane (Attane), then injected with 100 μL of the Nanoluc substrate (Promega) diluted 1:25 in sterile Hank’s balanced salt solution (HBSS; Gibco). Bioluminescence images were taken with the following settings: excitation block, emission 500 and 580 (20 nm bandpass filter), aperture f/1, exposure time 60 s, binning 8, and field of view 22.5 cm. All mice were imaged within 1 min upon injection with the substrate.

### Image Analysis

Image analysis of all *in vitro* and *in vivo* data taken using the IVIS was performed using the ImageJ software. Briefly, each raw grayscale TIF file was first processed to remove the background using the “HiLo lookup table,” then by setting the “Auto” contrast. “Despeckle” and “Remove outliers” (set to radius 1.00 and threshold 1.00) were applied to remove the outlier pixels. The division function of the image calculator was then used to compute for the R_580__/__510_. “Despeckle” was again applied to the resulting image to remove the noise. For *in vitro* imaging of plated cells, the mean IVIS R_580__/__510_ taken over several weeks was averaged and plotted out against the buffer of known pH level. For the *in vivo* images, R_580__/__510_ was obtained for the control mice and pHLuc mice individually. All *in vivo* images were presented using the 16-color lookup table. To allow for comparison of a specific mouse across different time points, the same range of maximum and minimum R_580__/__510_ pixel values were set for the same mouse across different time points.

### Immunohistochemistry

Palpable SW982 tumors of around 1.5 cm^3^ in volume were obtained from the mice and fixed in 4% paraformaldehyde in phosphate-buffered saline (PBS) for 24–48 h. The tumors were dehydrated, embedded in paraffin, then sectioned into 5 μm-thick samples onto glass slides. Antigen retrieval was performed at around 90°C in 10 mM citrate buffer (pH 6) for around 30 min. Three percent donkey serum in 2% BSA was used as a blocking solution, and samples were subsequently stained with anti-GFP antibody (Abcam) and anti-tRFP antibody (Evrogen), both diluted to 1:500 with blocking buffer. Samples were probed with the appropriate secondary antibodies anti-goat conjugated to AlexaFluor 488 and anti-rabbit conjugated to TRITC, respectively, diluted to 1:400. After washing the samples three times with PBS, mounting medium and a cover slip was placed over the samples. Images were then taken using the Axio Observer inverted microscope (Zeiss) under phase contrast as well as eGFP, dsRed, and Dapi filters to verify the presence of the fluorescent proteins.

## Data Availability Statement

The datasets generated for this study are available on request to the corresponding author.

## Ethics Statement

The animal study was reviewed and approved by the NUS Institutional Animal Care and Use Committee.

## Author Contributions

TO and ZA designed and performed the experiments and drafted the manuscript. RV and RK performed some experiments and contributed to manuscript writing. JT was involved in discussions. JD provided overall supervision of the project and writing of the manuscript.

## Conflict of Interest

The authors declare that the research was conducted in the absence of any commercial or financial relationships that could be construed as a potential conflict of interest.
