# Supplementary figures and images for: pHLuc, a Ratiometric Luminescent Reporter for in vivo Monitoring of Tumor Acidosis
Source: Front Bioeng Biotechnol. 2020 May 8;8:412. doi: 10.3389/fbioe.2020.00412 (PMC7225611; doi:10.3389/fbioe.2020.00412)

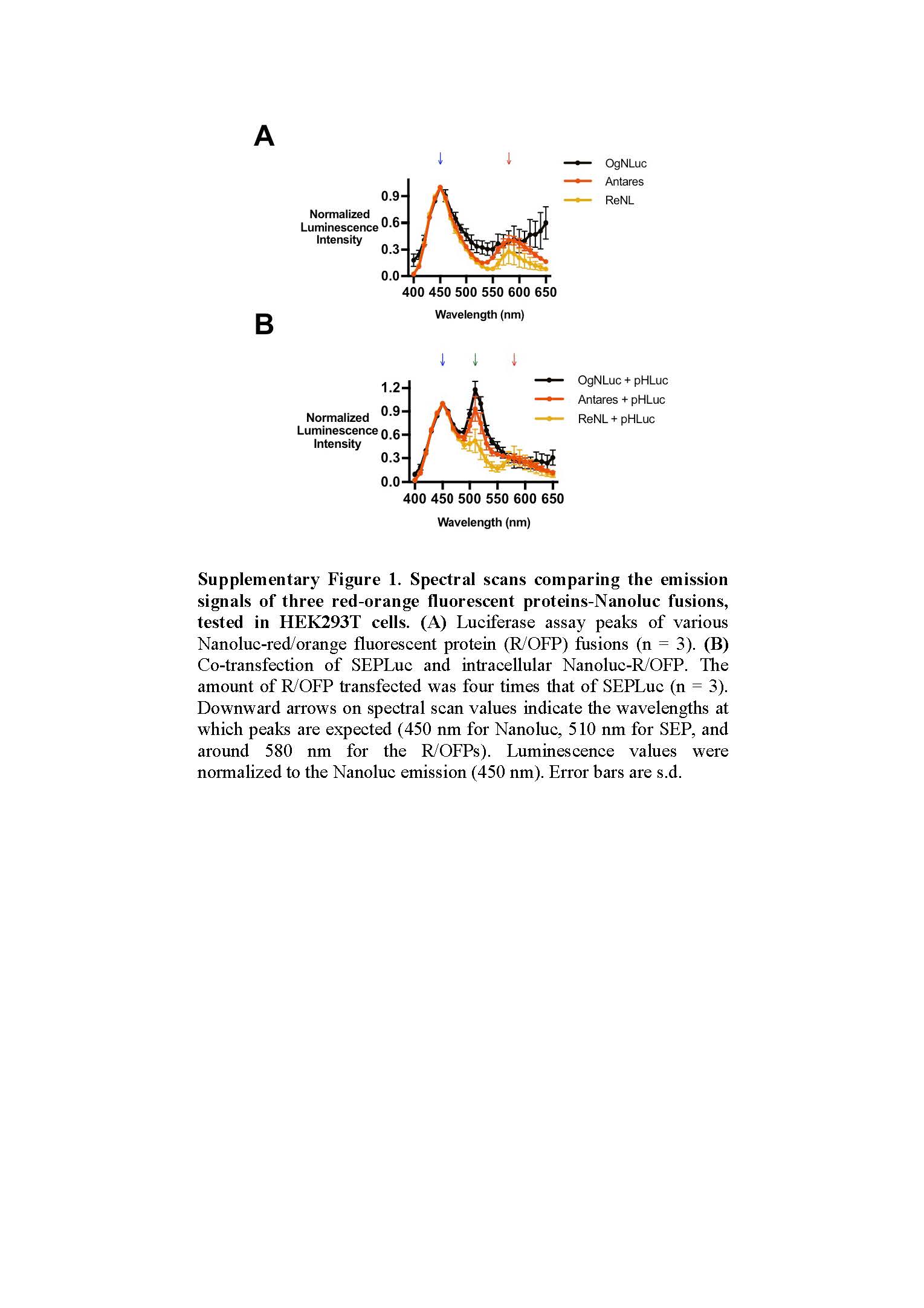

Supplement: Supplementary file 1 [file Image_1.JPEG]

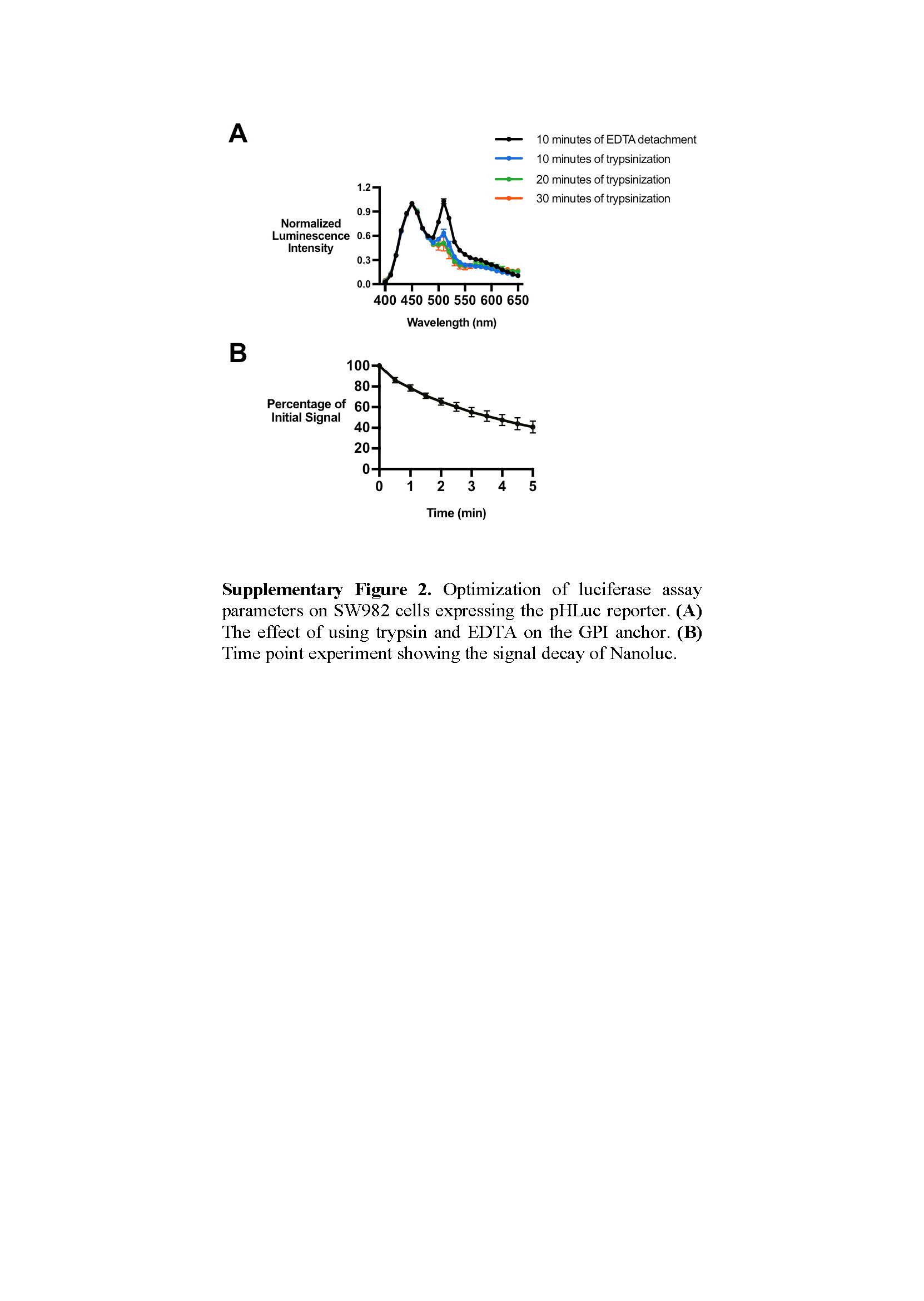

Supplement: Supplementary file 2 [file Image_2.JPEG]

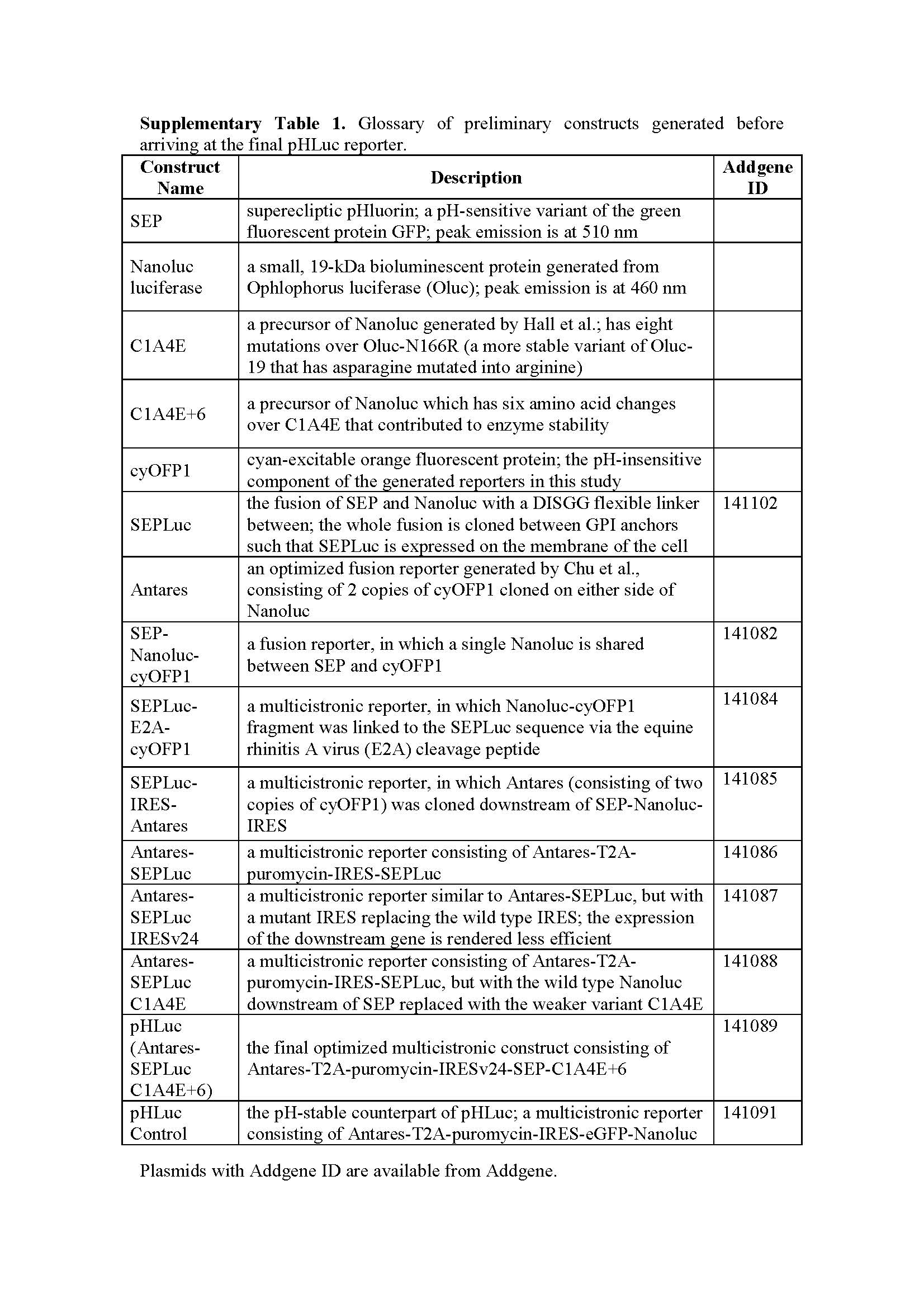

Supplement: Supplementary file 3 [file Image_3.JPEG]
